# Improving empathy and self-efficacy in caregivers of persons with intellectual disabilities, using m-learning (HiSense APP-ID): study protocol for a randomized controlled trial

**DOI:** 10.1186/s13063-018-2772-7

**Published:** 2018-07-27

**Authors:** Evelien van Wingerden, Paula S. Sterkenburg, Mirjam Wouda

**Affiliations:** 1 Department of Clinical Child and Family Studies, Vrije Universiteit, Van der Boechorststraat 1, 1081 BT Amsterdam, The Netherlands; 2 Stichting Ons Tweede Thuis, Hortensialaan 55A, 1431 VA Aalsmeer, The Netherlands; 3 Department of Psychotherapy, Bartiméus, Doorn, The Netherlands

**Keywords:** Attachment, Empathy, Self-efficacy, M-learning, Intellectual disability

## Abstract

**Background:**

A secure attachment with one or more caregivers is one of the most important predictors of cognitive development and emotional wellbeing. Persons with intellectual disabilities (ID) have extra need for secure relationships with primary caregivers but can find making connections difficult. This study aims to explore the effect of a non-invasive m-learning intervention on the empathy and self-efficacy of caregivers, in view of improving attachment relationships with persons with ID.

**Methods:**

A randomized controlled trial (RCT) will be conducted to assess the efficacy of the HiSense APP-ID. The study will include 100 parents/relatives and 100 professional caregivers of adults with mild or moderate ID (18 years and older). Of both groups of participants, half will complete the m-learning intervention. Data will be collected before the intervention starts, immediately after completion of the m-learning, and 1 month after the intervention. Participants will complete questionnaires concerning knowledge about attachment theory, empathy and self-efficacy (primary outcome measures) and social validity (secondary measures).

**Discussion:**

The intervention aims to increase caregiver understanding of attachment theory and to improve empathy and self-efficacy, which may lead to better care and less stress in social interactions. The HiSense APP-ID is an m-learning intervention that can be done independently on any digital device. The course is therefore easily accessible for caregivers of persons with ID. The current study will provide insight into the effectiveness of the intervention for parents/family members and professional caregivers of persons with mild or moderate ID.

**Trial registration:**

Nederlands Trial Register, NTR 6944. Registered on 16 December 2017.

**Electronic supplementary material:**

The online version of this article (10.1186/s13063-018-2772-7) contains supplementary material, which is available to authorized users.

## Background

A secure attachment with one or more caregivers is one of the most important factors in cognitive and emotional development. Persons with intellectual disability (ID) may remain dependent on a caregiver for various aspects of their lives, often including coping with emotions and stress. It is therefore crucial that parents and other caregivers of persons with ID are aware of their responsibilities in establishing a secure relationship and that they know how to support the emotional development of their loved one or client.

Children and their parents often form an attachment relationship, but other family members also can be involved, as can a caregiver at daycare or a teacher at school [1, 2]. The attachment figure alternately serves as a secure base and a safe haven: by functioning as a secure base, the attachment figure encourages a child to explore the world within safe boundaries. By providing a safe haven, the attachment figure gives the child assurance of available comfort in moments of distress [3–5]. A well-balanced presentation of these two aspects will support the emotional development of the child. Furthermore, the quality of an attachment relationship greatly depends on a caregiver’s sensitivity for the communicative signals of the other person. Equally important is an adequate response to these signals [4–6]. Depending on the sensitivity and responsiveness of the attachment figure, the child develops a general internal working model regarding confidence in the caregivers [7]. A child with secure attachments feels safe and confident in the interaction with others [8], and conversely, insecure attachment may lead to problematic coping strategies in children [9]. Parents and other caretakers are essential in teaching the child to apply appropriate coping strategies. Children with insecure attachment tend to display problematic behavior within social relationships and in dealing with stressful situations in daily life [10, 11].

Persons with ID may continue to need this type of guidance throughout their lives. Difficulties in information processing can present an obstacle to understanding social situations. Consequently, persons with ID are more likely to encounter situations they find overwhelming and stressful and in which they need attachment figures to assist in emotion regulation [9]. At the same time, parents and other caregivers may experience more difficulties in establishing a secure relationship with a child or adult with ID than with a typically developing person [12]. Janssen, Schuengel, and Stolk have listed four relevant main factors that can increase risk of insecure attachment [9]. First, parents may have difficulty accepting their child’s diagnosis, causing stress and limiting their sensitivity to any signals of stress or discomfort. Second, the communication skills of persons with ID are generally weak, which can lead to misinterpretation of their signals and behavior. Third, persons with ID may struggle to master the skills that are typically considered prerequisites for developing an attachment relationship, such as object permanence and distinguishing cause and effect. Fourth, persons with ID will often encounter a variety of caretakers at home and school and during other daily activities, which can be a source of stress. Some children and many adults with ID spend time in residential care where the availability of caretakers is limited or irregular, which is disruptive for the attachment relationship with their parents or other primary caregivers [13, 14].

Sensitivity and responsiveness correlate with an ability to understand someone else’s needs and emotions [15–17]. Empathy, which can be defined as the ability to tune into how the other person is feeling [18], is crucial in the development of a secure attachment style in children. Empathic behavior in parents is positively related to the attachment security and emotional openness of children [19]. Moreover, recent research by Settipani and Kendall [20] showed that higher levels of empathy allow mothers to estimate their child’s ability to face feared situations at a particular moment, a skill that is crucial in discerning when the child benefits most from a safe haven (providing comfort in times of distress) or a secure base (encouraging independence). Among medical professionals, empathy is positively linked to treatment outcomes, but this population shows a decline in empathy over time [21, 22].

In addition, self-efficacy can be an important factor in the behavior of parents and professional caregivers towards a person with ID [23]. For parents, a major source of experienced stress is their assessment of the situation and the various available support resources [24]. Parents who feel in control of the situation and are confident in their own parenting skills report lower stress levels than parents who do not feel that they can influence their child’s behavior [25]. A high level of parental stress may undermine parent sensitivity and responsiveness towards their son or daughter. Studies have shown that interventions based on attachment theory can boost self-efficacy in parents and induce attachment-related behavior between parents and their child [26, 27].

In short, improving the empathy and self-efficacy of caregivers may reduce stress in the home environment and, as a result, boost the cognitive and emotional functioning of the child. Various interventions have been developed to optimize attachment relationships between children and caregivers [26–29]. Bakermans-Kranenburg, van IJzendoorn, and Juffer [30] found that short interventions with a clear behavioral focus on enhancing parental sensitivity were the most successful in improving secure attachment in infants. Furthermore, Schuengel, De Schipper, Sterkenburg, and Kef [31] conclude in a summary of available research that interventions focusing on insight into attachment behaviors and relationships are most likely to stimulate the development of attachment relationships in persons with ID.

### Rationale

The present m-learning intervention aims to educate parents and caregivers and encourage reflection on the interaction with their adult child or client. The intervention aims to increase empathy by providing new information or refreshing their memory about the inner world of persons with ID. In addition, participants learn how to improve their sensitivity and responsivity through theoretical insights and practical examples. An increase in theoretical and practical knowledge enhances awareness in caregivers of their role in establishing a secure relationship with their child or client and increases their sense of self-efficacy.

The present intervention offers small quantities of theoretical and practical knowledge in an m-learning course that requires only 5 min each day over a period of 30 days. Each day, participants answer four multiple-choice questions and receive feedback. This approach allows active involvement in the learning process by giving the user the opportunity to think about answer alternatives. At the same time, the questions can be answered quickly enough to fit in the busy schedule of parents and professional caregivers.

In short, the study aims to explore the effects of the HiSense APP-ID intervention on the empathy and self-efficacy of parents/relatives and professional caregivers of persons with mild or moderate ID.

### Hypotheses

The intervention is hypothesized to have positive effects on the basic knowledge about attachment theory and to increase empathy and self-efficacy in parents/relatives and professional caregivers of persons with mild or moderate ID.

#### Primary hypotheses

The primary hypotheses are:

- Participation in the intervention will be associated with an increase in theoretical knowledge about sensitive and responsive interaction, for both parents/relatives and professional caregivers.
- Participation in the intervention will be associated with an increase in empathy and self-efficacy, for both parents/relatives and professional caregivers.

#### Secondary hypothesis

The secondary hypothesis is that parents/relatives and professional caregivers will experience the intervention as a pleasant and challenging way to gain theoretical knowledge.

## Methods

### Study design

**Fig. 1:**
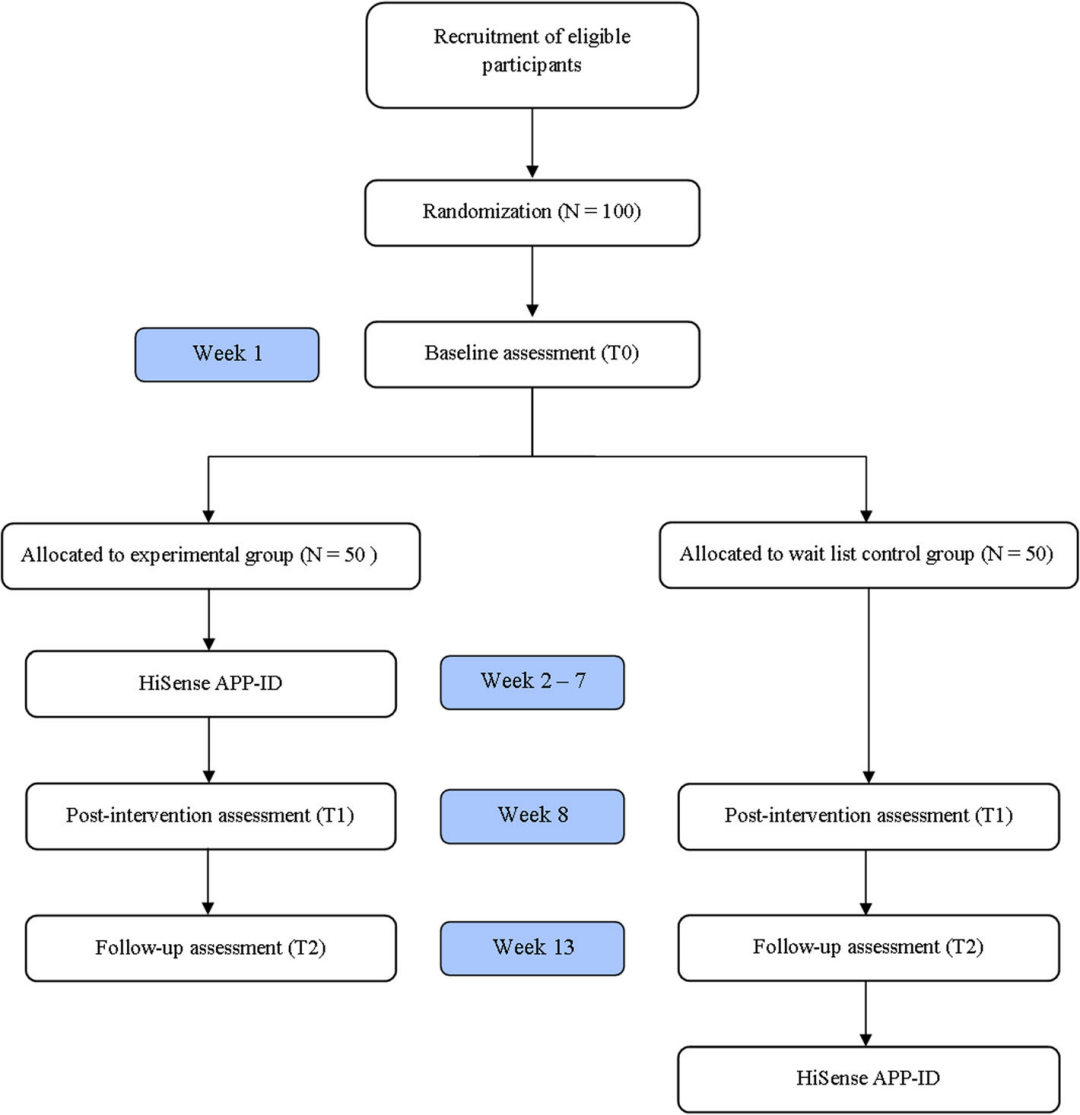
Timeline of the randomized controlled trial. This procedure will be followed separately for the two groups (parents/relatives and professional caregivers)

The effect of the HiSense APP-ID will be assessed in two separate two-group, parallel, single-blinded randomized controlled trials (Fig. 1). Parents and close family members are treated as one group and professional caregivers as another group. Each group will be analyzed separately.

We report the methods of this study according to the Standard Protocol Items: Recommendations for Interventional Trials (SPIRIT) Checklist (see Additional file 1).

**Fig. 2:**
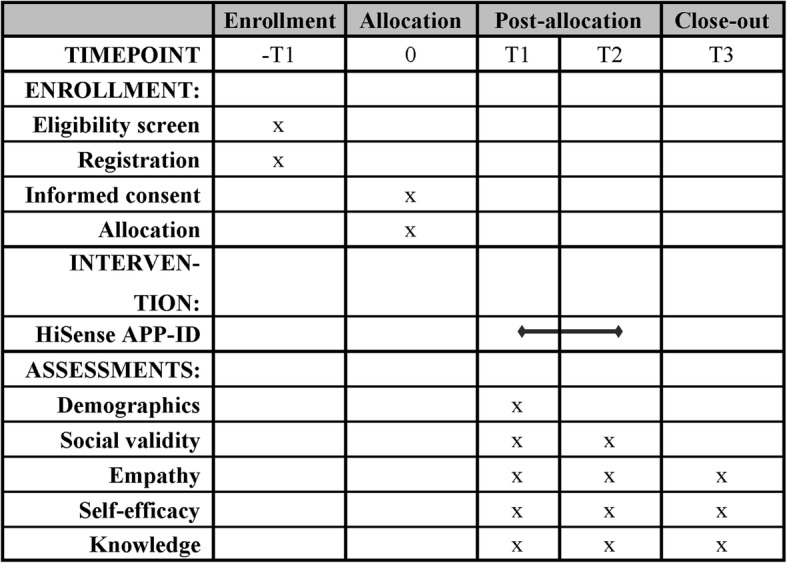
Schedule of enrollment, allocation, intervention, and assessments

Questionnaires on empathy and self-efficacy will be completed at three time points: at T0, before the intervention starts; at T1, when the intervention is finished; and at T2, when a retention measurement will be taken 30 days after the intervention (Fig. 2).

### Sample size

The sample size is estimated for linear mixed effects modeling with two conditions, three repeated measures, and clustered data. Based on a pilot study among caregivers in a care center, means and standard deviations can be estimated for empathy, self-efficacy, and knowledge. The desired power is set to a minimum of 0.90 and α = .05. The three care centers are taken into account as clusters with intra cluster correlation of .2. We estimate that around 90 participants are needed to detect a main effect of 0.5 SD on the primary outcome measures. To correct for attrition, we increased the highest number by 10%, bringing the total number of participants to 100 parents/relatives and 100 professional caregivers.

### Recruitment

The study will include two samples. The participants will be 100 professional caregivers and 100 parents/relatives of adults with mild or moderate ID (intelligence quotient (IQ) 35–70). This also included persons with ID and additional psychiatric diagnoses such as autism spectrum disorders, or additional physical disabilities such as a visual impairment. The group of professional caregivers will be recruited within three Dutch centers providing care for persons with ID living in group homes. Each care center has multiple group homes and activity centers in The Netherlands. The group of parents/relatives of adults with mild or moderate ID will be recruited from these and other centers throughout The Netherlands. Close relatives, such as a brother, sister, uncle, or aunt, can participate if the parents of a client are no longer the primary caregivers.

Participants in both samples will be recruited and enrolled by the participating care centers, which combined are connected to 3107 professional caregivers and 4830 parents/relatives. Participants will be invited to participate when they care for one or more persons with mild or moderate ID.

Candidates will be excluded from participation when they primarily care for persons with severe intellectual disability (IQ below 35) or when their use of HiSense APP-ID will be interrupted for more than five consecutive days. For parents/relatives, only one family member per client is included.

### Randomization

Cluster randomization among locations of the care centers will be used to prevent crossover effects between professionals and parents/relatives. Group homes and activity centers will be randomly assigned to the treatment condition or the care-as-usual condition by an independent party, based on order of entry and a pre-determined order of treatment conditions. Randomization of participants will occur after enrollment but before the baseline assessment. Participants are to be informed of their allocated condition shortly after T0 to reduce any response bias during the baseline assessment.

### Blinding

The research team will remain blind to the assigned condition of participants. Blinding participants to treatment condition will no longer be feasible after T0, when the assignment to conditions is revealed.

### Intervention

The HiSense APP-ID uses questions, statements, and practical examples to inform parents/relatives and professional caregivers about the influence of their sensitivity and responsivity in the context of attachment and communication. The m-learning intervention is divided into short, daily sessions. In addition to the advantage of taking little time, this approach is beneficial for the learning process. The topics are revisited repeatedly over time in different questions, leading to durable embedding in memory [32–34].

The main topics in the intervention are (1) attachment theory in daily practice, (2) socio-emotional functioning in persons with ID, (3) sensitivity and responsiveness to communicative signals, (4) emotion regulation, (5) observation and interpretation of behavior, and (6) basic knowledge about ID and common comorbidities such as autism spectrum disorder and attention deficit hyperactivity disorder. Each topic is addressed in 15 questions, except for attachment theory, which has 45 questions. Phrasing is differentiated for parents/relatives and professional caregivers, corresponding to their relationship to individuals with ID. For example, a question for professional caregivers is, “What does your client need to form a secure attachment relationship?” while for parents/relatives, the question is, “What does your child need to form a secure attachment?”

The intervention was developed in collaboration with healthcare professionals and parents/relatives of persons with mild and moderate ID. An advisory board of healthcare professionals was first consulted to determine the main topics of our first draft. The items were then judged by parents and professional caretakers on three points: (1) clarity of the questions, (2) clarity of the explanation, and (3) perceived relevance of the information. A total of 14 professional caretakers individually judged 12 items at random. Their comments were incorporated into the final revision of the content.

The structure of the intervention follows the first three categories of Bloom’s taxonomy of educational objectives ([35]; see [36] for adaptations): remember (acquisition of knowledge), understand (processing the content at a surface level), and apply (executing, implementing). The first 10 days (questions 1– 40) focus on background knowledge. On days 11–20 (questions 41–80), this knowledge is explicitly linked to practice, and on days 21–30 (questions 81–120), the items contain more elaborate examples and require deeper knowledge of the theory. Higher-level processing and application (the stages analyze, evaluate, and create) must then follow in daily practice.

### Measures

#### Demographics (T0)

At T0, the participants will answer questions on demographic characteristics, such as age, sex, level of education, work experience, and the relationship to the person with ID.

#### Empathy (T0, T1, T2)

The Empathy Quotient (EQ) questionnaire [18] (Dutch translation by De Corte and Uzieblo [37], adapted by Volman [38]), contains 60 items with a 4-point Likert scale. Participants indicate their level of agreement with statements regarding their sensitivity in social contexts. This study will use the abridged version of 40 items [18]. The EQ has high test–retest validity (*r* = .97, *p* < .001) and high internal consistency [39] (α = .92).

The Interpersonal Reactivity Index [40] (IRI) contains 28 items, divided over four subscales (perspective taking, fantasy, empathic concern, and personal distress). Participants indicate on a 5-point Likert scale how well each statement describes them. All subscales have sufficient internal reliability (.71–.77) and test–retest reliability (.62–.72).

#### Self-efficacy (T0, T1, T2)

The Self-Efficacy in the Nurturing Role scale ([41], translated [42]) is used to measure experienced competence of the participants. Participants answer 16 items about their role and experienced competence as a caregiver. On a 4-point Likert scale, respondents indicate to what extent the statements describe them. The scale has high internal consistency [41].

#### Knowledge (T0, T1, T2)

A short test of 25 multiple choice questions has been developed parallel to the development of the intervention. For each of the six main topics, one question is asked at the level of remembering, two questions assess understanding, and one question tests the ability to apply knowledge in a practical context. One additional question has been added to each test that requires applied knowledge of attachment theory in daily practice. Questions are similar to items in the intervention and differ at each measurement.

#### Social validity (T0, T1)

Participants will complete the Social Validity Scale [43] in the version as described by Janssen et al. [44] and Jonker et al. [45]. The questionnaire contains 32 items with a 5-point Likert scale on their expectations for the HiSense APP-ID (T0) or their experiences (T1) in terms of its practical use, their motivation for pursuing the intervention, and perceived effects on the quality of their care for their family member or client.

### Procedure

Participants will be recruited by the participating care centers. First, the centers will acquire groups of professional caregivers who work together in group homes or activity centers for persons with mild or moderate ID. Next, parents/relatives of clients will be approached for participation. If needed, recruitment for parents/relatives can be extended to other group homes and other care centers. All participants will be asked to give active consent for participation.

The study will contain three time points of data collection (Fig. 2): before the start of the intervention (T0), at the end of the intervention (40 days after T0), and 30 days after T1. For professional caregivers, researchers will make an appointment at the residence and provide a laptop to complete the questionnaires. Family members can come to the care center or complete the questionnaires online at home. Each measurement will take 45 min.

After T0, participants in the intervention condition will be asked to use the HiSense APP-ID for a total of 30 days. Each day, participants will complete four multiple-choice questions, which takes a maximum of 5 min per day. They will receive feedback on their original answer (correct or wrong) and an additional explanation for the correct answer. Participants do not have to log in on weekends, but after 2 days of inactivity, they are reminded of their participation by an automatic email. If a participant has not been active for 5 days, that person is excluded from further participation. There are 120 questions in total, which should be completed within 40 days. At the end of day 10 and day 20, participants can revisit the questions that were answered incorrectly. Participants in the “waiting list condition” receive care as usual.

At 40 days after T0, the knowledge tests and questionnaires are completed again by participants in both conditions (T1, see Fig. 1). After a retention period of 30 days, the questionnaires are completed for the last time (T2). Participants in the care-as-usual condition are then encouraged to use the app.

### Data recording

Privacy of the participants will be guaranteed by the use of personal identification numbers, to be assigned by a contact at the care centers. An email address is used only to send reminders during the intervention and to send a certificate of participation after completion of the intervention. Personal information about potential and enrolled participants will be kept within the organizations. Researchers will not see the names of the participants, and no detailed information will be obtained from the participants.

Questionnaires will be presented to participants in the digital Qualtrics web environment, which is accessed with a personal code. A guideline for using this program has been issued by the university’s ethical committee, which will be followed. Participants log into Qualtrics using only their identification number. To ensure anonymity of the participants, the program will not register the internet protocol (IP) address. No research data will appear on paper, and only the principal researchers will have access to the digital data. The data are stored according to the guidelines of the American Psychological Association until 10 years after publication of the results.

### Data management and monitoring

All data will be collected through computerized assessments, which obviate the need for double data entry. A data monitoring committee has not been established, because of minimal risks for the participants involved. A data management plan has been submitted to and accepted by the funding organization of the study (ZonMw), project number 845004004. The funding organization will conduct yearly site visits to check on the progress of the project.

This study is embedded in the Amsterdam Public Health (APH) research institute. The quality committee of APH has created an electronic quality assurance handbook to unify the conduct and safeguard the quality of research within the institute. In addition, APH randomly audits all projects to assure their quality. This study adheres to the quality handbook and anticipates the possibility of being randomly audited.

### Data analysis

All statistical analyses will be conducted using SPSS version 23 [46]. Both groups of participants (parents/relatives and professionals) will be treated as separate groups in the analyses because we expect that matching will not be possible. Partial intention-to-treat analyses will be performed: participants will be excluded from the analysis if fewer than two data points are available. All other participants will be analyzed according to their assigned condition.

For social validity, empathy, and self-efficacy, average item scores will be reported for each questionnaire. For the knowledge test, the total number of correct answers is to be used. Reliability statistics (Cronbach’s alpha) will be reported for the knowledge test at T0, T1, and T2.

Descriptive statistics will summarize the characteristics of the participants. Demographic variables are recorded as categorical variables, so chi-square analyses will be used to determine whether there are statistical differences between participant groups in the experimental and control condition at T0.

Increases in knowledge, empathy, and self-efficacy will be assessed using linear mixed effects modeling. Condition will be entered as a fixed effect and intercepts for participant and care center as random effects. Demographic variables will be included as a covariate in the analysis if significant between-group differences are found at T0.

## Discussion

Persons with ID have extra need for secure relationships with primary caregivers but can find making connections difficult. This study aims to explore the effect of a non-invasive m-learning intervention on the empathy and self-efficacy of caregivers, in view of improving attachment relationships with their family member or client with ID.

The m-learning is designed to teach or remind parents/relatives and caregivers of the importance of secure attachment relationships and to improve awareness of the perspective of their child or client. By increasing caregiver understanding of these topics, we expect that empathy and self-efficacy may grow, leading to better care and less stress in social interactions. To maximize the possible effect, the content was compiled in collaboration with parents/relatives and care professionals. The HiSense APP-ID is an m-learning intervention that can be done independently on any digital device and takes up little time. Thus, the intervention is suitable for adding to existing educational programs or as a periodic refreshment course for professional caregivers. The course is also easily accessible for family members of persons with ID. Furthermore, repeated exposures to information over a period of time can improve long-term learning gains [34].

At the same time, it remains to be seen whether independent participants will commit for the full duration of the course of 30 days or more. Automatic reminders will be sent to motivate participants, but some additional extrinsic motivation may be needed for them to finish the course, such as discussing the information with co-workers or with their partner. In addition, the content of the course is strictly theoretical despite its practical examples and recommendations. Participants must relate this information to daily practice and correctly apply their knowledge in new situations. In the case of questions, they may require the advice of a psychologist or behavioral scientist, such as a clinician at the care facility to which they are connected.

A practical concern may be that participants are not blind to their condition, which may cause bias in their responses on the questionnaires during the second measurement. However, test leaders are unaware of the condition of participants.

Because of a close collaboration with three large Dutch care centers in this project, the intervention can be implemented quickly if it proves effective. From the researchers’ point of view, the collaboration is helpful in offering the intervention to the target audience. From the parent and caregiver point of view, it is advantageous to be offered an evidence-based intervention that makes scientific literature on attachment theory available to them in small, practical portions.

In conclusion, the HiSense APP-ID can be a useful instrument for improving attachment relationships between persons with mild or moderate ID and their caregivers. Increased knowledge with regard to attachment theory may improve self-efficacy and empathy in parents/relatives and professional caregivers. Improving their basic knowledge may reduce stress and prevent the development of problematic behavior arising from a lack of experienced security in persons with ID.

## Trial status

Protocol version 1, dated 5 January 2018. The intervention has been developed. Recruitment of participants started within the participating care centers in October 2017, and data collection is planned to start in February 2018.

## Additional file

Additional file 1: SPIRIT 2013 Checklist. (DOC 121 kb)
